# Phosphorylation by Casein Kinase 2 Facilitates Psh1 Protein-assisted Degradation of Cse4 Protein*[Fn FN2]

**DOI:** 10.1074/jbc.M114.580589

**Published:** 2014-09-02

**Authors:** Geetha S. Hewawasam, Mark Mattingly, Swaminathan Venkatesh, Ying Zhang, Laurence Florens, Jerry L. Workman, Jennifer L. Gerton

**Affiliations:** From the ‡ Stowers Institute for Medical Research, Kansas City, Missouri 64110 and; § Department of Biochemistry and Molecular Biology, University of Kansas Medical Center, Kansas City, Kansas 66160

**Keywords:** Centromere, E3 Ubiquitin Ligase, Phosphorylation, Proteolysis, Yeast, CK2, Cse4/CENP-A, Psh1

## Abstract

Cse4 is the centromeric histone H3 variant in budding yeast. Psh1 is an E3 ubiquitin ligase that controls Cse4 levels through proteolysis. Here we report that Psh1 is phosphorylated by the Cka2 subunit of casein kinase 2 (CK2) to promote its E3 activity for Cse4. Deletion of *CKA2* significantly stabilized Cse4. Consistent with phosphorylation promoting the activity of Psh1, Cse4 was stabilized in a Psh1 phosphodepleted mutant strain in which the major phosphorylation sites were changed to alanines. Phosphorylation of Psh1 did not control Psh1-Cse4 or Psh1-Ubc3(E2) interactions. Although Cse4 was highly stabilized in a *cka2*Δ strain, mislocalization of Cse4 was mild, suggesting that Cse4 misincorporation was prevented by the intact Psh1-Cse4 association. Supporting this idea, Psh1 was also stabilized in a *cka2*Δ strain. Collectively our data suggest that phosphorylation is crucial in Psh1-assisted control of Cse4 levels and that the Psh1-Cse4 association itself functions to prevent Cse4 misincorporation.

## Teaser

**Background:** Psh1 is an E3 ubiquitin ligase that controls CenH3/Cse4 levels through proteolysis in budding yeast.

**Results:** Psh1 is phosphorylated *in vivo* by CK2, and its E3 ligase activity is promoted.

**Conclusion:** Phosphorylation is crucial in Psh1-assisted control of Cse4 levels, and the Psh1-Cse4 association itself functions to prevent Cse4 misincorporation.

**Significance:** This work reports a previously unknown function of CK2 in CenH3/Cse4 regulation.

## Introduction

Faithful chromosome segregation during cell division is crucial for the equal distribution of genetic material. Centromeric chromatin specifies the site of assembly of the kinetochore, a massive, evolutionarily conserved protein complex that attaches chromosomes to microtubules for segregation. In contrast to the complex regional centromeres found in higher eukaryotic organisms, budding yeast has a simple point centromere (1) containing a centromeric DNA (CEN)^2^ sequence of 125 bp (2, 3). A centromeric histone H3 variant, collectively known as CenH3 and in budding yeast as Cse4, replaces histone H3 in centromeric nucleosomes. Exclusive incorporation of CenH3 (or Cse4 in budding yeast) at the centromere is the foundation for proper kinetochore assembly, microtubule attachment, and chromosome segregation. Therefore, regulating Cse4 levels and deposition is a critical process. An increased dosage of Cse4 leads to mislocalization, kinetochore defects, and chromosome loss in budding yeast (4, 5). High levels of the human CenH3, CENP-A, result in misincorporation at non-centromeric sites (6). Overexpression of CENP-A leads to chromosomal instability in pRb-depleted human cells (7) and also causes CENP-A mistargeting and aneuploidy in colorectal cancer cells (8). Therefore, levels of CenH3 need to be carefully regulated.

Several factors regulate CenH3 proteins. Ubiquitin-mediated proteolysis controls Cse4 protein levels in budding yeast (9) and centromere identifier (CID), the CenH3 in *Drosophila* (10). Psh1 is an E3 ubiquitin ligase for Cse4 in budding yeast (11, 12). Psh1 controls Cse4 levels via ubiquitylation and proteolysis, preventing mislocalization of Cse4. In *Drosophila*, a multisubunit RING type E3 ubiquitin ligase, SCF^Ppa^, regulates centromere identifier (CID) proteolysis (13). Scm3, a centromeric nucleosome assembly factor, protects Cse4 from degradation possibly through binding to the CENP-A targeting domain of Cse4. However, additional mechanisms may contribute to Cse4 stability and deposition. Post-translational modifications (PTMs) of the enzymes involved in the ubiquitin-proteasome pathway for Cse4 could play a role in Cse4 regulation.

Phosphorylation can regulate ubiquitylation-coupled proteolysis at several levels. Both E3 ubiquitin ligase activity and E2 ubiquitin conjugating activity can be regulated via phosphorylation (14–16). In this study, we report the first evidence that phosphorylation regulates Psh1-assisted degradation of Cse4. Psh1 was phosphorylated primarily by the Cka2 subunit of casein kinase 2 (CK2). Our results suggest that phosphorylation of Psh1 by CK2 promotes its E3 activity toward Cse4. Although deletion of *CKA2* stabilized Cse4 more than deletion of *PSH1*, ectopic incorporation of Cse4 occurred at a lower level, suggesting that Psh1-Cse4 association itself prevents Cse4 misincorporation. Altogether, this study further advances our knowledge of how Cse4 is regulated in budding yeast.

## EXPERIMENTAL PROCEDURES

### 

#### 

##### Yeast Strains

All yeast strains are listed in supplemental Table S1.

##### Psh1-TAP and Dsn1-FLAG Purifications

The Psh1-TAP purification method was adopted from Puig *et al.* (17). The protocol for kinetochore purification using Dsn1-FLAG was adopted from Akiyoshi *et al.* (18).

##### Whole Cell Extract Co-immunoprecipitation (Co-IP)

Cell lysates were prepared in lysis buffer (50 mm Tris (pH 7.5), 150 mm NaCl, 0.1% Nonidet P-40, 1 mm DTT, 10% glycerol, and protease inhibitors). Protein concentration was determined using the Bradford assay. Cell lysates were diluted with dilution/wash buffer (50 mm Tris (pH 7.5), 150 mm NaCl, and 0.1% Nonidet P-40) and incubated with the antibody overnight at 4 °C. Prewashed Protein G Dynabeads were added and incubated for 2h at 4 °C. The beads were washed three times with dilution/wash buffer, and proteins were eluted with SDS buffer (10 mm Tris (pH 7.5), 1 mm EDTA, and 1% SDS). Immunoprecipitates were subjected to SDS-PAGE and Western blotting. Some co-IPs were performed using antibody-conjugated beads.

##### Proteasome Inhibition

Cultures grown to midlog phase in appropriate media were treated with MG132 (100 μm) or DMSO for 2–3 h. Cells were pelleted, washed with PBS, frozen in liquid N_2_, and stored in −80 °C.

##### Polyubiquitylated Protein Pulldown

Cell lysates were prepared in lysis buffer (same buffer used in co-IP with 15 mm *N*-ethylmaleimide), and protein concentration was measured using the Bradford assay. Polyubiquitin affinity resin (Thermo, 89899) (30 μl of a 25% slurry) was added to 2 mg of total proteins diluted 1:1 with TBS and incubated for 3 h or overnight at 4 °C. The beads were washed three times with wash buffer (lysis buffer:TBS, 1:9). Beads were boiled with 30 μl of SDS sample buffer, and samples were subjected to SDS-PAGE and Western blotting.

##### SDS-PAGE/Phos-tag-SDS-PAGE and Western Blotting

SDS-PAGE was performed using 4–12% precast gels (Invitrogen) or homemade 6% gels. For Phos-tag-SDS-PAGE, gels were prepared including acrylamide-pendant Phos-tag ligand (AAL-107). After running the gels, proteins were transferred onto PVDF membrane (Millipore, Immobilon-P), and Western blotting was performed according to a standard protocol.

##### Antibodies/Conjugated Beads

The antibodies used are as follows: anti-Myc (Covance, MMS150P), anti-Myc-HRP (Roche Applied Science, 11814150001), anti-HA (Covance, PRB101P, Roche Applied Science, 11867423001), anti-HA-HRP (Roche Applied Science, 12013819001), anti-ubiquitin (Covance, MMS257P), anti-Cse4 (polyclonal rabbit antibody against recombinant Cse4), anti-Pgk1 (Invitrogen, 459250), anti-FLAG (Sigma, F3165), anti-TAP (Open Biosystems, CAB1001), and anti-FLAG beads (Sigma, F2426).

##### Protein Stability Assay

Cells were grown to midlog phase in appropriate media. If Gal induction was needed, galactose was added to a final concentration of 4%, and cells were incubated for another 2 h. Cycloheximide (Sigma) was added to a final concentration of 10 μg/ml, and culture fractions were harvested at different time points. Cell lysates were prepared in lysis buffer, and protein concentration was determined using the Bradford assay. Proteins were analyzed by SDS-PAGE and Western blotting using the same amount of total proteins from each time point.

##### In Vitro Kinase Assay

Purified FLAG-His_6_-Psh1 (1 μg) was mixed with CK2 (1000 units) and 200 μm γ-labeled ATP (600 μCi/μmol) in a 50-μl final volume of CK2 reaction buffer (20 mm Tris-HCl, 50 mm KCl, and 10 mm MgCl_2_ (pH 7.5)). The reaction mixture was incubated at 30 °C for 2.5 h and boiled with SDS sample buffer. Proteins were separated using a 4–12% gel, and the gel was exposed to autoradiography film.

##### ChIP-Quantitative PCR

Cultures grown to an *A*_600_ of ∼0.7 were fixed in 1% formaldehyde for 15 min and then quenched with glycine (final concentration, 0.125 m) for 5 min. Cells were lysed for 1 h at 4 °C in FA-Lysis SDS buffer (50 mm Hepes (pH 7.5), 150 mm NaCl, 1 mm EDTA, 1% Triton X-100, 0.1% sodium deoxycholate, 0.2% SDS, and protease inhibitors). The lysate was sonicated using a Biorupter for 20 min (30 s on/off) at medium intensity. Protein concentration was determined, and samples were normalized before proceeding with the ChIP. 20% of total chromatin extract was used for IP using 2 μl of anti-Cse4 antibody and incubated at 4 °C overnight. 25 μl of Protein G Dynabeads (Invitrogen, 100-04D) prewashed in FA-Lysis buffer (50 mm Hepes (pH 7.5), 150 mm NaCl, 1 mm EDTA, 1% Triton X-100, 0.1% sodium deoxycholate) were added and incubated at 4 °C for 3 h. Beads were washed at room temperature with the following sequence of buffers; FA-Lysis buffer, FA-Lysis buffer with 1 m NaCl, FA-Lysis buffer with 500 mm NaCl, TEL buffer (0.25 m LiCl, 10 mm Tris-HCl (pH 8.0), 1 mm EDTA, 1% Nonidet P-40, and 1% sodium deoxycholate), and twice with 1× Tris-EDTA. Chromatin fragments were eluted twice using 200 μl of Elution Buffer (1% SDS, 250 mm NaCl, and 1× Tris-EDTA) at 65 °C with agitation. Two elutions were combined, treated with Proteinase K for 1 h at 55 °C, and incubated overnight at 65 °C. DNA was extracted with phenol-chloroform and precipitated using 100% ethanol. 2% of the total chromatin extract was processed for the input sample. Cse4 ChIPs were performed in triplicate with two no-antibody controls for each strain. Quantitative PCR was performed in triplicate for each sample using a Quanta Biosciences Perfecta SYBR Green FastMix.

##### Recombinant Protein Expression and Purification

The method for FLAG-His_6_-Psh1 expression and purification has been published (11). Recombinant CK2 was purchased from New England Biolabs (catalog number P6010L).

## RESULTS

### 

#### 

##### Psh1 Is Phosphorylated in Vivo

**FIGURE 1.**
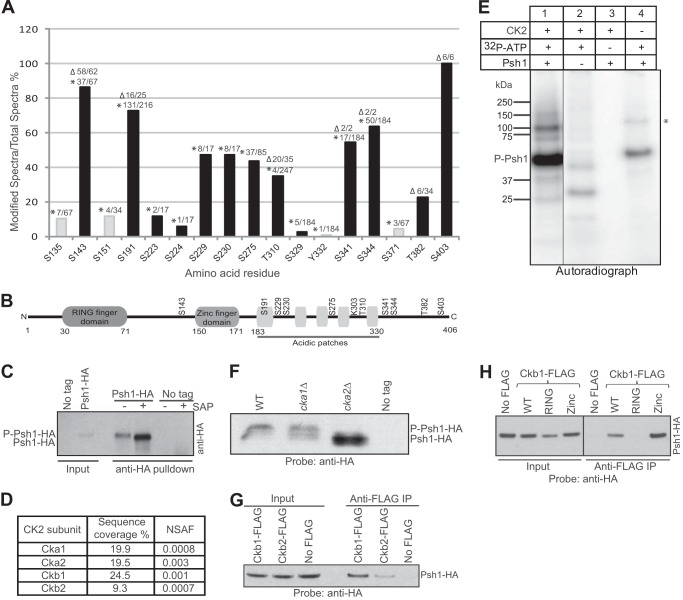
**Psh1 is phosphorylated *in vivo* by CK2.** *A*, PTM analysis using MS after affinity purification of Psh1-TAP and Dsn1-FLAG reveals phosphorylation sites of Psh1. The combined percentages of modified spectra/total spectra of the two purifications are graphed. The number of modified peptides/total peptides detected in Psh1-TAP (*) and Dsn1-FLAG (▵) samples are indicated above each *bar*. CK2 sites predicted by NetPhosK are in *black. B*, the major phosphorylation sites of Psh1 are shown relative to the three defined domains of a tripartite motif protein: RING finger motif, Cys_4_ zinc finger motif, and an acidic stretch. Lys-303 is the autoubiquitylation site of Psh1 identified by *in vitro* ubiquitylation and MS. *C*, Psh1-HA was immunoprecipitated from cell lysates using anti-HA, and eluates were subjected to shrimp alkaline phosphatase (*SAP*) treatment. Samples were analyzed by Phos-tag-SDS-PAGE (12% gel with 10 μm Phos-tag) and Western blotting with anti-HA antibodies. *D*, Psh1-TAP purification brings down all four subunits of CK2 as detected by MS. The normalized spectral abundance factor (*NSAF*) shows the estimated relative protein abundance calculated as described previously (38). *E*, Psh1 can be phosphorylated *in vitro* using recombinant CK2. Recombinant proteins and γ-labeled ATP were used to carry out a kinase assay *in vitro*. An autoradiograph following SDS-PAGE is shown. The band indicated by an *asterisk* could be a dimer of Psh1. *F*, the Cka2 catalytic subunit of CK2 is necessary for phosphorylation of Psh1. Strains expressing Psh1-HA were constructed bearing deletions in *CKA1* or *CKA2*. After Phos-tag-SDS-PAGE (7.5% gel with 10 μm Phos-tag) of cell lysates, Western blotting was carried out using anti-HA antibodies. The differences in band intensities in *C* and *F* could be due to the inefficient transfer of phosphorylated proteins from the Phos-tag gel to the membrane due to high affinity toward the Phos-tag. *G*, the CK2 regulatory subunits Ckb1 and Ckb2 pull down Psh1. Affinity-tagged strains (Psh1-HA/Ckb1-FLAG and Psh1-HA/Ckb2-FLAG) were used in co-IP. A strain lacking the tag on Ckb1 and Ckb2 was used as the control. In anti-FLAG co-IP using cell lysates, both Ckb1 and Ckb2 pull down Psh1. Ckb1 shows a much stronger interaction with Psh1 as revealed by a more intense band. *H*, Ckb1 binds to Psh1 via its RING finger domain. Strains were constructed expressing the affinity-tagged proteins Psh1-HA and Ckb1-FLAG. A co-IP using anti-FLAG antibody from whole cell lysates shows that the RING finger domain of Psh1 is necessary for Ckb1 interaction. A control IP was performed from a strain expressing Psh1-HA but lacking a FLAG tag on Ckb1. *P-Psh1*, phosphorylated Psh1.

How Psh1 E3 ligase activity is controlled is unknown, but we suspected that PTMs played a role. To investigate whether PTMs could regulate Psh1 activity, we affinity-purified Psh1-TAP and analyzed samples using mass spectrometry (MS) for PTMs. We were able to identify multiple phosphorylation sites in Psh1 (Fig. 1*A*). The Psh1-TAP purification is enriched mainly for the soluble pool of Psh1. To specifically examine centromere-associated Psh1, we purified kinetochore proteins using Dsn1-FLAG, a subunit of the kinetochore MIND complex (18), and analyzed PTMs of the Psh1 in these samples by MS. We identified the same phosphorylation sites in centromere-associated Psh1 as found in Psh1-TAP samples as well as two additional sites (Fig. 1*A*). Major phosphorylation sites with >20% of modified/total spectra are indicated in Fig. 1*B* relative to the major protein domains in Psh1. To further confirm the phosphorylation of Psh1, we used an epitope-tagged Psh1-HA strain (Fig. 1*C*). Psh1-HA was immunoprecipitated from whole cell extracts, and eluates were subjected to shrimp alkaline phosphatase treatment to remove phosphate groups from phosphorylated proteins. Samples were analyzed by Phos-tag-SDS-PAGE and Western blotting with anti-HA antibodies. On a Phos-tag gel, phosphorylated proteins migrate more slowly compared with lesser or non-phosphorylated counterparts. We detected a faster migrating Psh1-HA band in the shrimp alkaline phosphatase-treated sample compared with the untreated sample, further confirming *in vivo* phosphorylation of Psh1.

##### CK2 Is a Kinase for Psh1

CK2 is a highly conserved serine/threonine kinase in eukaryotic organisms. This versatile kinase controls many important cellular processes such as signal transduction, transcription, translation, metabolism, and cell cycle progression. Hundreds of CK2 substrates have been identified so far (19). CK2 is a heterotetrameric enzyme composed of two catalytic and two regulatory subunits. In budding yeast, the catalytic subunits are Cka1 and Cka2, and the regulatory subunits are Ckb1 and Ckb2. Most of the Psh1 phosphorylation sites identified by MS are predicted to be CK2 sites by NetPhosK, a program for prediction of potential kinases. These potential CK2 sites are depicted in *black bars* in Fig. 1*A*. In addition, all four subunits of CK2 co-purified with Psh1-TAP (Fig. 1*D*), further suggesting CK2 as a potential kinase for Psh1.

To determine whether CK2 is a kinase for Psh1, we performed a kinase assay *in vitro* utilizing recombinant Psh1, CK2, and radiolabeled ATP (Fig. 1*E*). The autoradiograph clearly shows phosphorylated Psh1 in the presence of both CK2 and ATP, suggesting that Psh1 is a substrate for CK2. Recombinant Psh1 used in this assay was purified using a baculovirus expression system in insect cells. The lower intensity phosphorylated Psh1 band observed with the control sample without CK2 (Fig. 1*E*, *lane 4*) is possibly due to some kinase activity that co-purified with Psh1.

To identify which subunit of CK2 is responsible for phosphorylating Psh1, we deleted each catalytic subunit (*cka1*Δ or *cka2*Δ) in the strain expressing Psh1-HA. Single deletions were made because double deletion of the two catalytic subunits is reported to be lethal (20). Cell lysates of the mutants were compared with WT using Phos-tag-SDS-PAGE and Western blotting with anti-HA antibodies (Fig. 1*F*). Psh1-HA in the *cka1*Δ strain migrated similarly to WT, whereas in the *cka2*Δ strain it showed a faster migrating, lesser or non-phosphorylated band, suggesting that Cka2 is primarily responsible for Psh1 phosphorylation.

To identify the subunits of CK2 that are physically interacting with Psh1, epitope-tagged strains with Psh1-HA/Ckb1-FLAG and Psh1-HA/Ckb2-FLAG were used to perform co-IP and Western blotting (Fig. 1*G*). We were able to detect both Ckb1 and Ckb2 interacting with Psh1. However, the interaction between Psh1 and Ckb1 was much stronger. These data demonstrate that CK2 is a kinase for Psh1 with catalysis and interaction primarily mediated by Cka2 and Ckb1, respectively.

Psh1 contains a RING domain, a type of zinc finger that can bind two zinc cations, and a zinc finger domain (Fig. 1*B*). The RING domain is essential for the Psh1-Cse4 interaction, and a mutation in the RING domain stabilizes Cse4 (11). We tested the RING and zinc finger mutants for interaction with Ckb1 of CK2 and Ubc3/Cdc34, the E2 ubiquitin-conjugating enzyme for Psh1 (see comment in Hewawasam *et al.* (11)). Co-IP showed that the Ckb1-Psh1 interaction required the RING finger domain of Psh1 (Fig. 1*H*). The RING domain appears to be required for Psh1 to interact with both Cse4 and Ckb1. However, neither the RING nor zinc finger domain was required for the Psh1-Ubc3 interaction (data not shown). This suggests that the interaction between Psh1 and Ubc3 is facilitated by an unidentified region of Psh1.

##### CK2 Is Important for Efficient Regulation of Cellular Cse4 Levels

**FIGURE 2.**
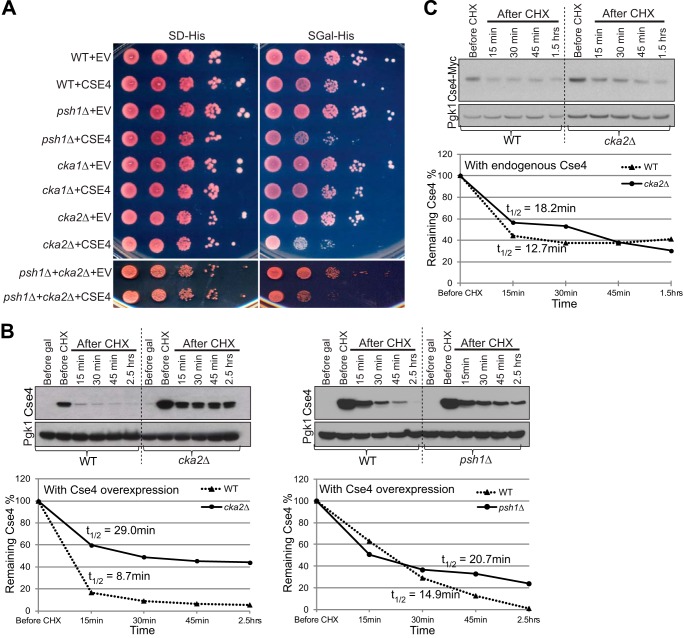
**The catalytic subunit Cka2 of CK2 is important for efficient regulation of Cse4 levels.** *A*, Cse4 was overexpressed from the *GAL* promoter on a 2-μm plasmid in WT, *psh1*Δ, *cka1*Δ, *cka2*Δ, and *psh1*Δ*cka2*Δ strains. *EV* indicates an empty vector control as a point of comparison. 10-Fold serial dilutions of overnight cultures were plated to either SD-His or Gal-His medium. All strains grow similarly on SD-His but *psh1*Δ, *cka2*Δ, and *psh1*Δ*cka2*Δ strains overexpressing Cse4 grow poorly. *B*, deletion of *CKA2* stabilizes Cse4. WT, *psh1*Δ, and *cka2*Δ strains from *A* were used to perform a Cse4 ubiquitylation assay. After Gal induction of Cse4 overexpression, CHX was added, and cells were collected at the time points indicated for analysis by Western blotting using an anti-Cse4 antibody. *C*, Cka2 regulates endogenous levels of Cse4. Cse4-Myc was expressed from the native Cse4 promoter. Levels of Cse4-Myc were measured in cell lysates at the time points indicated following CHX treatment in WT and *cka2*Δ strains. In *B* and *C*, Pgk1 was the loading control. The same amount of total protein was loaded per lane. Graphs show quantifications of the Cse4 bands normalized to Pgk1. Cse4 *t*_½_ was calculated by fitting normalized Cse4 band intensity data to a first-order decay function as explained in Belle *et al.* (39).

*PSH1* is not an essential gene, and a simple deletion of *PSH1* does not cause a defect in a minichromosome loss assay (11). Deletion of *PSH1* resulted in slow growth when Cse4 was overexpressed from the *GAL* promoter on a 2-μm plasmid. Cse4 overexpression in a *psh1*Δ strain alters kinetochore function as revealed by a delay in destruction of anaphase inhibitor Pds1 (12). If CK2 is important for Psh1-mediated Cse4 regulation, then overexpression of Cse4 in a CK2 mutant might be expected to show a growth phenotype similar to *PSH1* deletion, and Cse4 protein levels should be subsequently stabilized. To test this, we overexpressed Cse4 in catalytic subunit deletion mutants *cka1*Δ and *cka2*Δ (Fig. 2*A*). We observed slow growth with *cka2*Δ but not with *cka1*Δ (Fig. 2*A*, compare *cka1*Δ+*CSE4* and *cka2*Δ+*CSE4* with *psh1*Δ+*CSE4*), consistent with our observation that Cka2 was primarily responsible for phosphorylation of Psh1. Cse4 overexpression in the double mutant *psh1*Δ*cka2*Δ showed a growth phenotype similar to that of either single mutant, suggesting the Psh1 and Cka2 function in the same pathway to control Cse4 levels.

We then examined the Cse4 protein levels in these strains using a protein stability assay (Fig. 2*B*). After Gal induction of Cse4 overexpression, cycloheximide (CHX) was added to inhibit protein translation, and the level of Cse4 in cell lysates was determined at different time points using Western blotting with anti-Cse4 antibody. We observed highly stabilized Cse4 levels in a *cka2*Δ mutant compared with a WT strain, and the half-life of Cse4 (Cse4 *t*_½_) was about 3 times longer in a *cka2*Δ strain (Fig. 2*B*, *left panel*). Cse4 was more stabilized by *cka2*Δ than *psh1*Δ. We next measured the stability of endogenous levels of Cse4-Myc (Fig. 2*C*). Cse4-Myc was also stabilized in a *cka2*Δ strain compared with a WT strain. These results suggest that phosphorylation of Psh1 by CK2 may promote its E3 activity for Cse4. Because the two stability assays in Fig. 2*B* were performed separately, the difference in Cse4 stability in WT strain between the *left* and *right panels* could be due to fluctuations in the experimental conditions. However, the two stability assays with Cse4 overexpression resulted in an average Cse4 *t*_½_ of 12 min for WT strain, which is comparable with a Cse4 *t*_½_ of WT strain (12.7 min) with endogenous Cse4 levels in Fig. 2*C*. Conversely, in *cka2*Δ strain, Cse4 *t*_½_ was elevated with Cse4 overexpression (Fig. 2, compare *B*, *left panel*, with *C*; 29.0 *versus* 18.2 min, respectively). Assuming that the experimental conditions are comparable in Fig. 2, *B*, *left panel*, and *C*, a high level of soluble Cse4 and inefficient Cse4 degradation in the absence of Cka2 may elevate Cse4 *t*_½_ in the *cka2*Δ strain with Cse4 overexpression.

**FIGURE 3.**
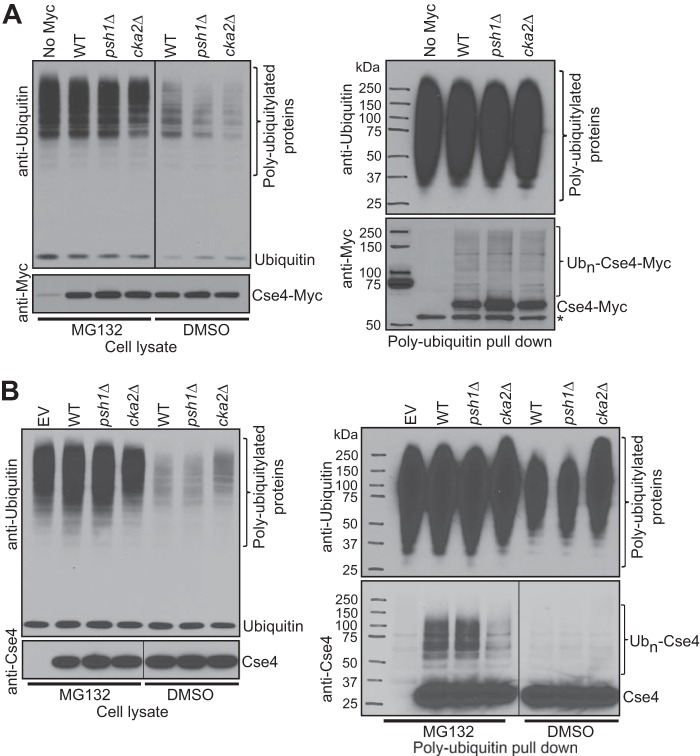
**Deletion of *CKA2* reduces cellular levels of polyubiquitylated Cse4.** *A*, levels of Ub*_n_*-Cse4-Myc were measured in WT, *psh1*Δ, and *cka2*Δ strains with a deletion in *PDR5.* Cse4-Myc was expressed from its native promoter. An untagged strain was used as a control. Cultures were grown to midlog phase and treated with either MG132 (100 μm) or DMSO for 3.5 h. Anti-ubiquitin and anti-Myc Western blots of cell lysates confirmed the efficiency of proteasome inhibition by MG132 and similar Cse4-Myc levels, respectively (*left panel*). Polyubiquitylated proteins were pulled down from 2 mg of total proteins (from MG132-treated samples) using polyubiquitin affinity resin. Final elutions were probed with anti-ubiquitin and anti-Myc antibodies after SDS-PAGE (*right panel*). No obvious difference was detected in Ub*_n_*-Cse4-Myc levels among WT, *psh1*Δ, and *cka2*Δ strains. A nonspecific band is marked with an *asterisk. B*, Ub*_n_*-Cse4 levels are reduced in the *cka2*Δ strain. WT, *psh1*Δ, and *cka2*Δ strains from Fig. 2*A* were used to perform a Cse4 ubiquitylation assay. *EV* indicates the empty vector control. After Gal induction of Cse4 for 2 h, cells were treated with either MG132 (100 μm) or DMSO for another 2 h. Anti-ubiquitin and anti-Cse4 Western blots of cell lysates confirmed the efficiency of proteasome inhibition by MG132 and similar Cse4 levels, respectively (*left panel*). Polyubiquitylated proteins were pulled down, and final elutions were analyzed as in *A* (*right panel*).

We next examined ubiquitylated forms of Cse4 in WT and *cka2*Δ strains with both endogenous Cse4 and overexpression (Fig. 3). Cells expressing Cse4-Myc at endogenous levels were grown to midlog phase and treated with MG132 for 2 h to inhibit proteasome function. Pdr5, a plasma membrane transporter, is deleted from these strains to improve MG132 uptake. The efficiency of proteasome inhibition was confirmed by high accumulation of polyubiquitylated proteins in MG132-treated cells compared with DMSO control samples (Fig. 3*A*, *left panel*, anti-ubiquitin Western blot). Anti-Myc Western blotting showed similar Cse4-Myc levels in the lysates (Fig. 3*A*, *left panel*). Polyubiquitylated proteins were pulled down from MG132-treated samples using polyubiquitin affinity resin, and eluates were probed with anti-ubiquitin and anti-Myc antibodies (Fig. 3*A*, *right panel*). Pulldown samples probed with anti-ubiquitin showed that the efficiency of enrichment of polyubiquitylated proteins was nearly the same. A *psh1*Δ strain was included as a control. We did not observe an obvious difference in polyubiquitylated Cse4-Myc (Ub*_n_*-Cse4-Myc) levels among WT, *cka2*Δ, and *psh1*Δ strains when Cse4-Myc was expressed at an endogenous level.

The Cse4 stability assay in Fig. 2*B* suggests that a reduction in the Ub*_n_*-Cse4 levels between WT and *cka2*Δ might be more obvious if Cse4 was overexpressed. Therefore, we examined Ub*_n_*-Cse4 levels in the strains in Fig. 2*B* (Fig. 3*B*). After Gal induction of Cse4, cells were treated with MG132. The method described in Liu *et al.* (21) was used to inhibit proteasome function using MG132. The efficiency of proteasome inhibition was confirmed by high accumulation of polyubiquitylated proteins in MG132-treated cells compared with DMSO control samples (Fig. 3*B*, *left panel*, anti-ubiquitin Western blot). Anti-Cse4 Western blotting showed similar Cse4 levels in the lysates (Fig. 3*B*, *left panel*). Polyubiquitylated proteins were pulled down as explained before, and samples were analyzed by Western blotting (Fig. 3*B*, *right panel*). The efficiency of enrichment of polyubiquitylated proteins in MG132-treated samples was very similar, and the signal was reduced in DMSO control samples. Ub*_n_*-Cse4 was detected only in MG132-treated samples, confirming that these bands are polyubiquitylated Cse4 normally targeted for proteasomal degradation. Under our experimental conditions, we could not observe an obvious difference in Ub*_n_*-Cse4 levels between WT and *psh1*Δ strains, suggesting that differences in ubiquitylation may be more difficult to detect than differences in protein stability. As expected, however, we detected a dramatic reduction in Ub*_n_*-Cse4 levels in a *cka2*Δ strain compared with WT. These results further suggest that CK2 promotes ubiquitylation and degradation of Cse4.

Cse4 is only partially stabilized in a *psh1*Δ strain, indicating the presence of additional factors controlling Cse4 levels *in vivo* (11, 12). Interestingly, Cse4 was more stabilized by *cka2*Δ than *psh1*Δ (Fig. 2*B*), suggesting that CK2 may control factors in addition to Psh1. Ubc3/Cdc34, the E2 ubiquitin-conjugating enzyme for Psh1, is regulated by CK2 phosphorylation (15, 16). CK2 phosphorylation within the N-terminal catalytic domain of Ubc3 up-regulates its ubiquitin charging activity (16). Therefore, in a *cka2*Δ mutant, ubiquitin charging of Ubc3 might be inefficient, causing extra stabilization of Cse4 levels. Recently, Fpr3 has been reported as another regulator of Cse4 stability (22). Fpr3 is also phosphorylated by CK2 (23). Moreover, there are several reports implicating CK2 in regulation of 26 S proteasome subunits via phosphorylation (24, 25). These reports, along with data from our studies, suggest that CK2 could regulate Cse4 levels via phosphorylation of Psh1, E2/Ubc3, other regulators of Cse4, and the proteasome. We continued to explore the role of CK2 phosphorylation in Psh1 activity in particular.

##### Phosphorylation of Psh1 Promotes Destruction of Cse4

**FIGURE 4.**
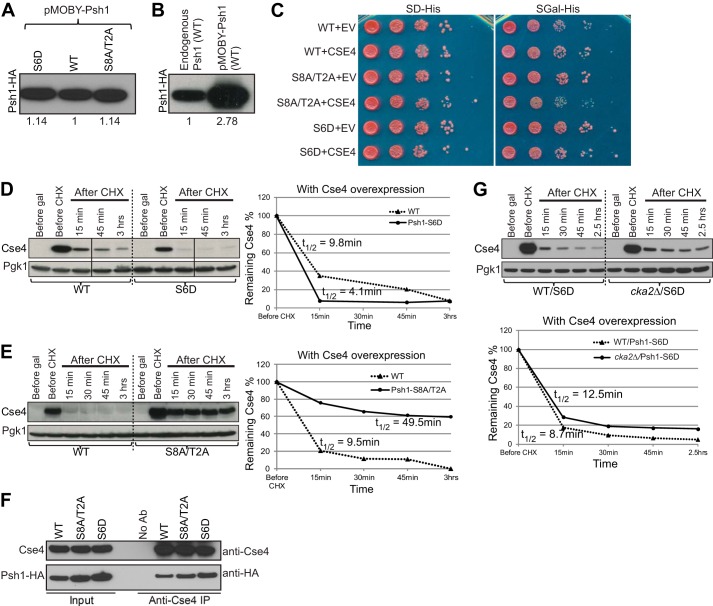
**Phosphorylation of Psh1 promotes its E3 activity for Cse4.** *A*, ectopic expression levels of HA-tagged WT, phosphomimic mutant (Psh1-S6D-HA), and phosphodepleted (Psh1-S8A/T2A-HA) Psh1 ectopically expressed on a plasmid were compared. Cell lysates probed with anti-HA antibody show very similar Psh1-HA levels. Intensities of the Psh1-HA bands relative to WT protein are indicated. *B*, ectopic expression level of WT Psh1-HA was compared with endogenous Psh1-HA (WT) expressed under the native promoter. Cell lysates probed with anti-HA antibody show an about 3 times higher Psh1-HA level after ectopic expression. *C*, a growth assay was performed using phosphomimic and phosphodepleted Psh1 strains. In these strains, the endogenous copy of *PSH1* is deleted, and Psh1-S6D or Psh1-S8A/T2A was ectopically expressed using a plasmid. Cse4 was overexpressed from the *GAL* promoter on a 2-μm plasmid. *EV* indicates an empty vector control as a point of comparison. 10-Fold serial dilutions of overnight cultures were plated to either SD-His or Gal-His medium. All strains grow similarly on SD-His, but the Psh1-S8A/T2A strain overexpressing Cse4 grows poorly. *D*, the stability of Cse4 in the presence of phosphomimic Psh1 mutant (Psh1-S6D) was measured using a protein stability assay. After 2 h of Cse4 induction, CHX was added, and cells were collected at the time points indicated for analysis by Western blotting using an anti-Cse4 antibody. Cse4 was destabilized in the presence of the phosphomimic Psh1 mutant. *E*, the stability of Cse4 in the presence of phosphodepleted Psh1 mutant (Psh1-S8A/T2A) was measured using a protein stability assay as in *D*. Cse4 was stabilized in the presence of the phosphodepleted Psh1 mutant. *F*, phosphodepleted and phosphomimic mutants of Psh1 interact with Cse4. Cse4 was overexpressed from the *GAL* promoter on a 2-μm plasmid in the presence of affinity-tagged Psh1-S6D-HA or Psh1-S8A/T2A-HA. Co-immunoprecipitation using anti-Cse4 antibody pulls down Psh1-HA from WT, Psh1-S6D-HA, or Psh1-S8A/T2A-HA strain. A control IP was performed without antibody. *G*, phosphomimic Psh1 mutant can bypass the effect of *CKA2* deletion on Cse4 stability. In these strains, the endogenous copy of *PSH1* is deleted, and Psh1-S6D was ectopically expressed using a plasmid. The stability of Cse4 was measured using a protein stability assay as in *D*. In the presence of the phosphomimic Psh1 mutant, Cse4 stabilization in a *cka2*Δ strain relative to WT is very mild compared with the stability observed in a *cka2*Δ strain in the presence of wild type Psh1 (see Fig. 2*B*, *left panel*). In *D*, *E*, and *G*, Pgk1 was the loading control. The same amount of total protein was loaded per lane. The graphs show quantification of the Cse4 bands normalized to Pgk1. Cse4 *t*_½_ was calculated as in Fig. 2. *Ab*, antibody.

Because CK2 can potentially control ubiquitin-mediated proteolysis of Cse4 at multiple levels, stabilization of Cse4 in a *cka2*Δ strain may not directly reflect how Psh1 phosphorylation by CK2 affects the stability of Cse4. Therefore, we wanted to examine how Psh1 phosphorylation affects Cse4 using phosphomimic and phosphodepleted Psh1 mutants. We identified 10 major *in vivo* phosphorylation sites of Psh1 using MS (Fig. 1, *A* and *B*). All these residues are predicted to be potential CK2 sites by NetPhosK. In the phosphomimic Psh1 mutant (Psh1-S6D), six of these phosphorylation sites, identified using Psh1-TAP purification, were changed to aspartic acids (S143D, S191D, S229D, S230D, S275D, and S344D) to mimic phosphate groups. In the phosphodepleted Psh1 mutant (Psh1-S8A/T2A), all 10 identified phosphorylation sites were changed to alanine (S143A, S191A, S229A, S230A, S275A, T310A, S341A, S344A, T382A, and S403A) to prevent phosphorylation of Psh1. WT or Psh1 mutants were ectopically expressed from a plasmid in a *psh1*Δ background. Psh1 protein levels expressed from the plasmid were very similar as confirmed using an HA affinity-tagged version of these plasmids (Fig. 4*A*). However, compared with endogenous Psh1 levels, ectopic expression from the plasmid gave about 3 times more Psh1 (Fig. 4*B*).

If phosphorylation of Psh1 is important for Cse4 regulation, then overexpression of Cse4 in the phosphodepleted Psh1 mutant might be expected to show a growth phenotype similar to that of *PSH1* and *CKA2* deletion, and Cse4 protein levels should be stabilized. To test this, we overexpressed Cse4 in the presence of either Psh1-S6D or Psh1-S8A/T2A mutant (Fig. 4*C*). As expected, we observed slow growth with the Psh1-S8A/T2A strain. We next examined Cse4 stability in these strains. Cse4 was destabilized in the Psh1-S6D strain (Fig. 4*D*) and was dramatically stabilized in the Psh1-S8A/T2A strain (Fig. 4*E*). We further performed a co-IP in the presence of affinity-tagged Psh1-S6D-HA or Psh1-S8A/T2A-HA overexpressing Cse4 (Fig. 4*F*). Both Psh1-S6D-HA and Psh1-S8A/T2A-HA interacted with Cse4. This confirms that the stabilization of Cse4 in the presence of Psh1-S8A/T2A mutant is not the result of poor association of Cse4 with the mutant but more likely due to inefficient E3 ligase activity of the mutant. These results suggest that phosphorylation of Psh1 by CK2 may promote the E3 ubiquitin ligase activity of Psh1 for Cse4. Further confirming our hypothesis, we did not observe substantial Cse4 stabilization in a *CKA2* deletion strain relative to WT in the presence of Psh1-S6D (Fig. 4*G*). The phosphomimic mutant of Psh1 bypassed the effect of *CKA2* deletion, and Cse4 was efficiently targeted for degradation (compare Fig. 4*G* with Fig. 2*B*, *left panel*). We did not observe an obvious Cse4 stabilization in a strain expressing the Psh1-S6A mutant, which is the phosphodepleted counterpart of Psh1-S6D (data not shown). Because the Psh1-S6A mutant still contains four major phosphorylation sites, lack of Cse4 stabilization in Psh1-S6A may suggest that Thr-310, Ser-344, Thr-382, and Ser-403 are some of the most important phosphorylation sites for activation of Psh1. These phosphorylation sites were detected mainly in centromere/kinetochore-bound Psh1 in the Dsn1-FLAG purification.

**FIGURE 5.**
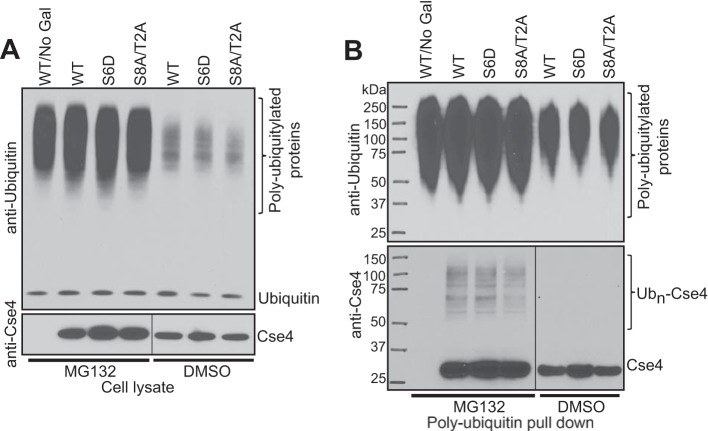
**Ub*_n_*-Cse4 levels are reduced in the presence of the phosphodepleted Psh1 mutant.** *A*, strains from Fig. 4 were used to measure polyubiquitylated Cse4 levels as in Fig. 3. A wild type strain without Gal induction (*WT/No Gal*) was used as a control. Anti-ubiquitin and anti-Cse4 Western blots of cell lysates confirmed the efficiency of proteasome inhibition by MG132 and similar Cse4 levels, respectively. *B*, polyubiquitylated proteins were pulled down, and final elutions were analyzed as in Fig. 3. An anti-Cse4 Western blot shows reduced levels of Ub*_n_*-Cse4 in the presence of the phosphodepleted Psh1 mutant.

Next, we looked at Ub*_n_*-Cse4 levels in the strains in Fig. 4, *D* and *E*, using the method explained earlier, polyubiquitylated protein pulldown and Western blotting (Fig. 5). Although we could not detect a difference in Ub*_n_*-Cse4 in the strain expressing Psh1-S6D compared with a strain with wild type Psh1, an obvious reduction in Ub*_n_*-Cse4 levels was observed between WT and the Psh1-S8A/T2A mutant (Fig. 5*B*). These results further support the idea that CK2 phosphorylation of Psh1 activates its E3 ubiquitin ligase activity for Cse4.

##### Phosphorylation of Psh1 by CK2 May Control Ubiquitin Transfer onto Cse4

**FIGURE 6.**
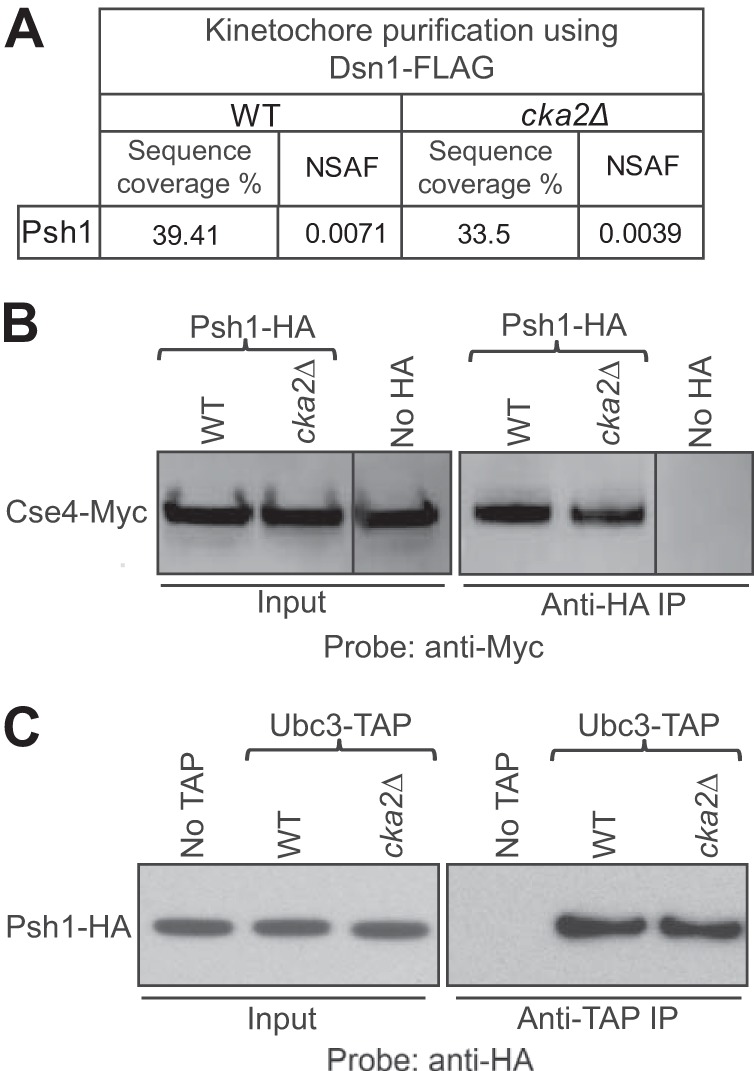
**The interaction of Psh1 with Cse4 and Ubc3 was unaffected by phosphorylation.** *A*, Psh1 co-purifies with kinetochores in a *cka2*Δ strain. Purification of kinetochores via Dsn1-FLAG followed by MS analysis shows Psh1 in both WT and *cka2*Δ strains. The normalized spectral abundance factor (*NSAF*) shows the estimated relative Psh1 protein abundance. *B*, Psh1-Cse4 interaction was unaffected by deletion of *CKA2*. Strains expressing Cse4-Myc/Psh1-HA were used for co-IP. A control IP was performed from a strain expressing Cse4-Myc but lacking Psh1-HA. Psh1-HA pulls down Cse4-Myc in lysates made from either WT or a *cka2*Δ strain. *C*, the interaction between Psh1 and Ubc3 was unaffected by deletion of *CKA2*. Strains expressing Ubc3-TAP/Psh1-HA were used for co-IP. A control IP was performed from a strain expressing Psh1-HA but lacking Ubc3-TAP. Ubc3-TAP pulls down Psh1-HA in lysates made from either WT or a *cka2*Δ strain.

During Cse4 ubiquitylation, Psh1 should act as an adaptor by recruiting Cse4, the substrate, and the ubiquitin-charged E2, Ub∼Ubc3, thereby facilitating ubiquitin transfer from Ubc3 to Cse4. Phosphorylation may control interactions of Psh1 with Cse4 and/or Ub∼Ubc3, ubiquitin transfer from Ub∼Ubc3 to Cse4, or all of these steps. We have reported previously that Psh1 interacts with Cse4 and localizes to centromeres/kinetochores (11). In an affinity purification of kinetochore-associated proteins using Dsn1-FLAG followed by MS analysis, we detected Psh1 in both WT and *cka2*Δ strains (Fig. 6*A*). Therefore, phosphorylation of Psh1 by CK2 may not be important for Psh1 interaction with Cse4 and centromere localization. Supporting this, we observed no change in Cse4 association with the Psh1-S6D-HA or Psh1-S8A/T2A-HA mutants (Fig. 4*F*).

To further investigate how CK2 phosphorylation affects the interaction of Psh1 with Cse4, we performed a co-IP (Fig. 6*B*). Psh1-HA pulled Cse4-Myc down to a similar level in both WT and *cka2*Δ, confirming that phosphorylation by CK2 is not necessary for Psh1-Cse4 interaction. In another co-IP, we observed comparable levels of Psh1-HA coming down with Ubc3-TAP in both WT and *cka2*Δ backgrounds, demonstrating that phosphorylation by CK2 is not necessary for Psh1-Ubc3 interaction (Fig. 6*C*). Therefore we reasoned that phosphorylation does not control the physical interactions between Psh1 and Cse4 or Psh1 and Ubc3 but rather that the ubiquitin transfer from Ub∼Ubc3 to Cse4 is controlled by phosphorylation. Supporting this idea, we detected reduced levels of Ub*_n_*-Cse4 in both a *cka2*Δ strain and the strain expressing phosphodepleted Psh1-S8A/T2A (Figs. 3*B* and 5). Ubiquitin charging of Ubc3 has been reported to be positively regulated by CK2 phosphorylation of Ubc3. Therefore, reduced levels of Ub*_n_*-Cse4 in the *cka2*Δ strain could be due to the collective effect of poor ubiquitin charging on Ubc3 and inefficient ubiquitin transfer from Ub∼Ubc3 to Cse4. Reduced levels of Ub*_n_*-Cse4 in the Psh1-S8A/T2A strain could reflect inefficient ubiquitin transfer from Ub∼Ubc3 to Cse4.

Recent work suggests a role for protein subunits of the Skp1-Cdc53/Cullin-F box (SCF) complex in centromere/kinetochore regulation. For example, a subunit of the Mis18 complex, which regulates CENP-A localization in human cells, is targeted for proteolysis by SCF (26). SCF is an evolutionarily conserved multisubunit E3 ubiquitin ligase (27). Interestingly, Ubc3, the E2 ubiquitin-conjugating enzyme for Psh1, functions as the E2 for SCF E3 ligases (28). This raises the question whether Psh1 functions as a subunit of the SCF complex to facilitate Cse4 proteolysis. The stability of centromere identifier (CID), *Drosophila* CenH3, is regulated by Ppa, which is an F box protein subunit of the SCF^Ppa^ E3 ligase complex (13). We tested whether Psh1 can interact with Cdc53, an invariant core subunit of SCF, using a co-IP. We did not observe any interaction (data not shown), suggesting that Psh1/Ubc3 acts on Cse4 independently of other SCF components.

**FIGURE 7.**
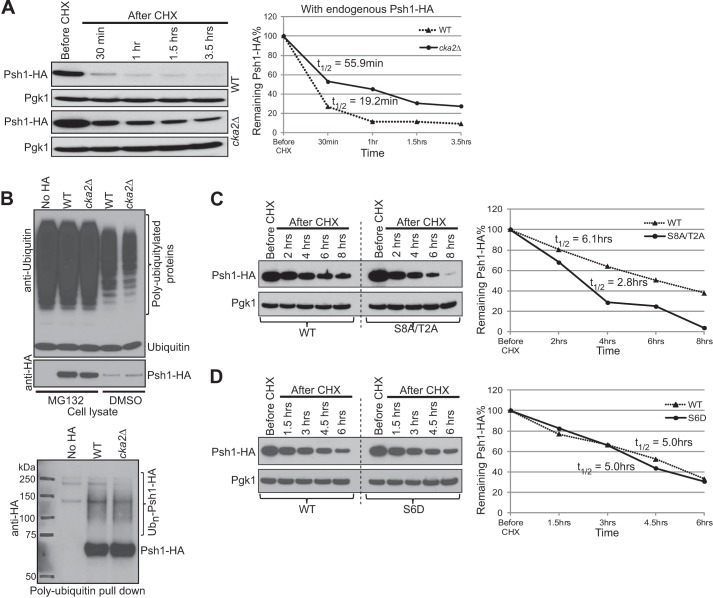
**Psh1 was stabilized in the absence of *CKA2*.** *A*, Endogenous levels of Psh1-HA were measured in cell lysates at the time points indicated, following CHX treatment in WT and *cka2*Δ strains. Pgk1 was the loading control. The same amount of total protein was loaded per lane. The graph shows quantification of the Psh1-HA bands normalized to Pgk1. Psh1 *t*_½_ was calculated by fitting normalized Psh1 band intensity data to a first-order decay function as in Fig. 2. *B*, changes in polyubiquitylated Psh1 levels *in vivo* are not detected in a *cka2*Δ strain. Psh1-HA was expressed from the endogenous promoter in WT and *cka2*Δ strains. An untagged strain was used as a control. Ub*_n_*-Psh1-HA levels were measured using the method in Fig. 3. Anti-ubiquitin and anti-HA Western blotting of cell lysates confirmed the efficiency of proteasome inhibition by MG132 and similar Psh1-HA levels, respectively (*upper panel*). No obvious difference was detected in Ub*_n_*-Psh1-HA levels between WT and *cka2*Δ strains (*lower panel*). *C*, the stability of the phosphodepleted mutant of Psh1 was measured. Psh1-S8A/T2A-HA was ectopically expressed using a plasmid in an endogenous *PSH1* deletion background. After addition of CHX, cells were collected at the time points indicated for analysis by Western blotting using anti-HA antibody. *D*, the stability of the phosphomimic Psh1-S6D-HA mutant of Psh1 was measured as in *C*. In *C* and *D*, Pgk1 was used as the loading control. The graphs show quantification of the Psh1-HA bands normalized to Pgk1. Psh1 *t*_½_ was calculated as in *A*.

We previously reported an autoubiquitylation site, Lys-303 (indicated in Fig. 1*B*), on Psh1 identified by *in vitro* ubiquitylation followed by MS (11). This indicates that Psh1 levels could also be controlled through proteolysis. *In vivo*, Psh1 ubiquitylation could be mediated by itself and by one or more other E3 ligases. Phosphorylation of Psh1 by CK2 may regulate ubiquitylation and proteolysis of Psh1 as well. To test this, we performed a Psh1 stability assay using a strain expressing Psh1-HA as the sole copy from the endogenous promoter (Fig. 7*A*). We observed stabilized Psh1 levels with *CKA2* deletion. We further examined Ub*_n_*-Psh1 levels *in vivo* (Fig. 7*B*). If CK2 phosphorylation of Psh1 facilitates ubiquitylation of Psh1, then Ub*_n_*-Psh1-HA should be diminished in a *cka2*Δ strain. Although Psh1 was stabilized in *cka2*Δ strain, we did not see a reduction in Ub*_n_*-Psh1-HA (Fig. 7*B*, *lower panel*). Therefore, phosphorylation of Psh1 by CK2 may not control ubiquitylation of Psh1, or the difference may be beyond the detection limit of this assay such as we observed with Cse4 ubiquitylation in the *psh1*Δ strain. Supporting the idea that phosphorylation may not facilitate ubiquitylation and degradation of Psh1, we did not observe a stabilization of Psh1-S8A/T2A-HA or destabilization of Psh1-S6D-HA compared with Psh1 (Fig. 7, *C* and *D*). In fact, the phosphodepleted mutant, Psh1-S8A/T2A-HA, was destabilized compared with WT (Fig. 7*C*). Although this mutant could associate with Cse4 to a similar level as wild type Psh1 (Fig. 4*F*), it might be recognized as a defective protein due to compromised E3 ligase function and targeted for faster degradation by cellular mechanisms.

##### Cse4 Is Mislocalized in a cka2Δ Strain

**FIGURE 8.**
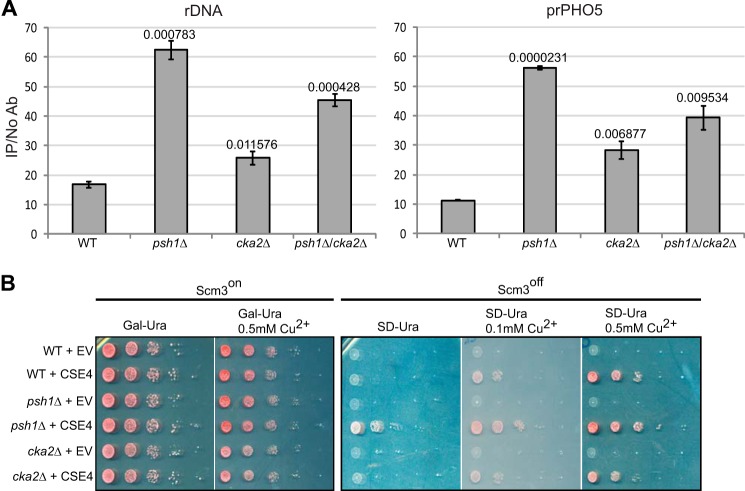
**Non-centromeric localization of Cse4 is increased in a *cka2*Δ strain.** *A*, ChIP was performed for Cse4 in strains overexpressing Cse4 from Fig. 2*A*. Quantitative PCR was used to detect Cse4 levels for the *PHO5* promoter and ribosomal DNA. The *y axis* indicates arbitrary units representing the enrichment for each sequence from ChIP performed with and without antibody with respect to the signal for total chromatin for each sample. The *error bars* represent the standard deviation for ChIP performed in triplicate. The same pattern of Cse4 localization in the *PHO5* promoter and ribosomal DNA was observed in a biological replicate, confirming the reproducibility of the results. *B*, deletion of *CKA2* does not improve growth of Scm3^off^ strain. Strains were constructed in which the only copy of Scm3 was under the control of the *GAL* promoter, allowing the expression of Scm3 to be controlled with glucose/galactose. Cse4 was under the control of a copper-inducible promoter on a 2-μm vector. *EV* indicates empty vector. When Scm3 is expressed (Scm3^on^), no growth differences are observed in 10-fold serial dilutions. Lower levels of induction of Cse4 (SD-Ura + 0.1 mm Cu^2+^) rescue Scm3 ^off^ when *PSH1* is deleted (*psh1*Δ) as compared with WT. *CKA2* deletion does not improve growth of Scm3^off^ under the same conditions. *Ab*, antibody.

Overexpression of Cse4 in a *psh1*Δ strain was toxic, and Cse4 was significantly mislocalized (Figs. 2*A* and 8*A* and Ref. 11). Because a *cka2*Δ strain showed high Cse4 levels, we predicted that Cse4 mislocalization would increase. We used Cse4 strains from Fig. 2*A* to examine Cse4 levels at non-centromeric regions. We followed Cse4 at the ribosomal DNA and *PHO*5 promoter using ChIP followed by quantitative PCR (Fig. 8*A*). Cse4 mislocalization to these regions was elevated in a *cka2*Δ strain compared with a WT strain. However, the mislocalization was significantly lower compared with a *psh1*Δ strain, although the overall level of Cse4 in the *cka2*Δ strain was higher than that observed in a *psh1*Δ strain. Cse4 mislocalization was increased with the *psh1*Δ*cka2*Δ double deletion compared with the *cka2*Δ single deletion. Both Cse4 and Psh1 were significantly stabilized in a *cka2*Δ strain (Figs. 2*B* and 7*A*), and Psh1-Cse4 interaction was not disrupted by the deletion of *CKA2* (Fig. 6*B*). Therefore, we suggest that the association of Cse4 with Psh1 may reduce non-centromeric deposition of Cse4 in the *cka2*Δ strain. Psh1-Cse4 association could reduce the amount of Cse4 available to the histone chaperones responsible for non-centromeric deposition. When Psh1 is removed in a *cka2*Δ strain, more Cse4 becomes available for chaperones, leading to increased misincorporation of Cse4.

Scm3 is a centromeric nucleosome assembly factor (29) and is essential to recruit Cse4 to centromeres and to maintain a functional kinetochore (30). Growth of a Scm3 deletion or shutoff (Scm3^off^) strain can be rescued by overexpression of Cse4, and deletion of *PSH1* improves rescue (11, 30). Under these conditions, some other histone chaperone must be responsible for the assembly of Cse4 nucleosomes. (For instance, the H3.3 chaperone death domain associated protein (DAXX) was reported to regulate ectopic localization of CENP-A in human cells (31).) If Psh1-Cse4 association prevents Cse4 availability for nucleosome assembly, then deletion of *cka2*Δ may not rescue the Scm3^off^ strain. To test this, we performed a growth assay using strains containing Gal-inducible endogenous Scm3 and copper-inducible Cse4 (Fig. 8*B*). The level of Cse4 can be controlled with copper concentration. Growth of WT, *psh1*Δ, and *cka2*Δ strains was compared under Scm3^on^ and Scm3^off^ conditions. We observed no growth difference between the strains when Scm3 was on. However, when Scm3 was off in the *cka2*Δ strain compared with the *psh1*Δ strain, rescue of growth by Cse4 overexpression was poor (Fig. 8*B*, Scm3^off^). This result is consistent with the notion that Cse4 availability for nucleosome assembly requires deletion of *PSH1*, and *CKA2* deletion does not suffice. Together, our data suggest that Psh1-Cse4 physical association itself functions to prevent mislocalization of Cse4.

## DISCUSSION

**FIGURE 9.**
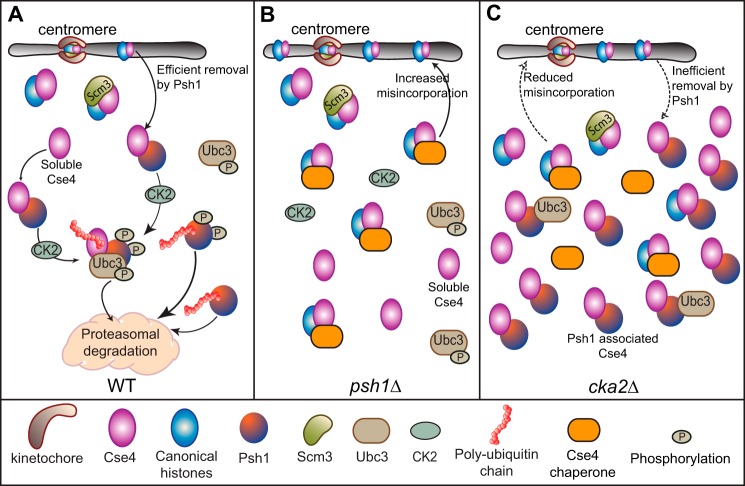
**Model for how Cse4 is controlled by Psh1 and CK2.** *A*, under normal conditions, CK2 phosphorylation activates Psh1 and Ubc3, facilitating Cse4 ubiquitylation and proteolysis. Phosphorylation of Psh1 is not required for the association of Psh1 with Cse4 or Ubc3; rather it facilitates ubiquitin transfer onto Cse4. Cse4 that is associated with the nucleosome assembly factor Scm3 is protected from Psh1. Soluble Cse4 and mislocalized Cse4 may also be removed by Psh1. CK2 helps to maintain Cse4 at the proper level via regulation of ubiquitin transfer by Ubc3/Psh1. *B*, when *PSH1* is deleted, the deficit in Psh1-mediated ubiquitylation of Cse4 causes accumulation of soluble Cse4. Chaperones can bind to and assemble Cse4 nucleosomes at non-centromeric locations. Without Psh1, mislocalized Cse4 is more difficult to remove. *C*, when *CKA2* is deleted, the lack of CK2 phosphorylation and activation of Psh1/Ubc3 causes stabilization of Cse4. Psh1 is also stabilized with *CKA2* deletion. Psh1 can still bind to Cse4, sequestering it from chaperones and preventing Cse4 incorporation into non-centromeric nucleosomes.

Here we report that phosphorylation by CK2 facilitates Psh1-assisted Cse4 degradation. Lack of complete stabilization of Cse4 following deletion of *PSH1* suggested additional regulation of Cse4 stability (11). We demonstrate that phosphorylation events by CK2 provide an additional mechanism for regulating Cse4 stability. Based on our findings, we propose a model for how Cse4 is controlled by Psh1 and CK2. In wild type cells (Fig. 9*A*), CK2 phosphorylates Psh1, activating the E3 ubiquitin ligase function of Psh1 toward Cse4. CK2 phosphorylation of Ubc3 also improves ubiquitin transfer from Ub∼Ubc3 to Cse4. Activation of Psh1 promotes efficient removal of the soluble pool of Cse4 and may also improve the removal of misincorporated Cse4. This regulation is especially important when Cse4 is overexpressed. In a *psh1*Δ strain (Fig. 9*B*), lack of Psh1-mediated proteolysis results in stabilization and misincorporation of Cse4. The histone chaperones responsible for the misincorporation of Cse4 remain to be identified. In a *cka2*Δ strain (Fig. 9*C*), the lack of Psh1 and Ubc3 phosphorylation dramatically stabilizes Cse4. However, the reduced Psh1/Ubc3 activity is not problematic because Psh1 is also stabilized and can act as an effective sink for excess Cse4, preventing Cse4 misincorporation.

We suggest that Psh1/CK2 may help to control both the centromeric and non-centromeric pools of Cse4. Two recent studies show that Pat1 localizes to centromeres and maintains a pool of Cse4 in the vicinity of the kinetochore cluster (32, 33). Centromere/kinetochore-bound Psh1 and CK2 may help regulate this accessory Cse4 pool. Psh1 may also target non-centromeric Cse4 with the help of the *facilitates chromatin transcription (*FACT) complex, a chromatin disassembly/reassembly factor (12). Psh1 was first discovered associated with Spt16 and Pob3, components of the budding yeast FACT complex. All four subunits of CK2 co-purify with Spt16 along with Psh1 (34), and the Cka2 subunit of CK2 associates with chromosome arms as revealed by ChIP (35). This suggests that CK2 phosphorylation could regulate Psh1 targeting of misincorporated Cse4 as well.

Regulation of kinetochore proteins by kinases/phosphatases is well known. The evolutionarily conserved kinase Ipl1/Aurora B regulates kinetochore function through phosphorylation of kinetochore proteins (36). Opposing activities of Mps1 and PP1 regulate the phosphorylation level of kinetochore protein Spc105 (37). Evidence from previous reports suggests that CK2 might regulate kinetochore function. Studies using a temperature-sensitive mutant *cka1*Δ *cka2-8* demonstrate that Cka2 functions in cell cycle progression (35). *cka1*Δ *CKA2* cells showed DNA content similar to wild type and normal mitotic spindle elongation and chromatid separation. However, *cka1*Δ *cka2-8* cells had abnormal DNA profiles, short spindles, and problems with sister chromatid separation at non-permissive temperature, suggesting an important role of Cka2 in kinetochore function and chromosome segregation. Interestingly, Mif2 and Ndc10 are targets of CK2 (35). Although Ipl1 and CK2 show an antagonistic effect on Mif2 stability, the two kinases have a synergistic effect on Ndc10. By analogy, CK2 may play an antagonistic/synergistic role with another kinase for Psh1. The phosphorylation of Psh1 may be regulated by additional phosphatases or deubiquitylases. In any event, our results continue to implicate CK2 as an important kinase regulating kinetochore-associated proteins.

Our work reported here identifies a previously unknown function of CK2 in Cse4 regulation. Our study lays the foundation for future work on the role of CK2 in proteolysis of Cse4 in budding yeast and potentially of CenH3 variants or additional kinetochore components in other eukaryotic organisms. Future studies will help unravel more details regarding the proteolytic surveillance mechanisms that regulate cellular Cse4 and facilitate faithful chromosome segregation.

## Supplementary Material

Supplemental Data

## Footnotes

2 The abbreviations used are:

CEN: centromeric DNA
CK2: casein kinase 2
CenH3: centromeric histone H3 variant
CENP-A: centromere protein A
SCF: Skp1-Cdc53/Cullin-F box
PTM: post-translational modification
TAP: tandem affinity purification tag
IP: immunoprecipitation
CHX: cycloheximide
Ub*_n_*-Cse4: polyubiquitylated Cse4
SD: synthetic defined.
