# Supplementary material for: Phosphorylation by Casein Kinase 2 Facilitates Psh1 Protein-assisted Degradation of Cse4 Protein
Source: J Biol Chem. 2014 Sep 2;289(42):29297–309. doi: 10.1074/jbc.M114.580589 (PMC4200280; doi:10.1074/jbc.M114.580589)
Supplement: Supplemental Data [file supp_289_42_29297__index.html]

Phosphorylation by Casein Kinase 2 Facilitates Psh1 Assisted Degradation of Cse4 — Phosphorylation by Casein Kinase 2 Facilitates Psh1 Protein-assisted Degradation of Cse4 Protein — Phosphorylation Facilitates Degradation of Cse4 — Supplemental Data 

# Phosphorylation by Casein Kinase 2 Facilitates Psh1 Protein-assisted Degradation of Cse4 Protein

## Supplemental Data

**Files in this Data Supplement:**

- Supplemental Table 1 (.xls, 40 KB) - Strains used in this study.
